# Conventional versus endoscopic-assisted crestal sinus lifting with simultaneous implant placement: a comparative clinical study

**DOI:** 10.1186/s40729-025-00653-3

**Published:** 2025-11-13

**Authors:** Samy Elian, Ashraf Abdelfattah, Abdelaziz Baiomy

**Affiliations:** 1 Head Oral Surgery Department, Sohag University, Sohag, Egypt; 2 Consultant OMFS Al-Azhar University, Assuit, Egypt

**Keywords:** Maxillary sinus augmentation, Posterior maxillary implants, Sinus endoscopy

## Abstract

**Patients:**

Twenty patients with residual bone heights of 4–7 mm in the posterior maxilla were randomly divided into two groups: Group 1 (conventional crestal approach) and Group 2 (endoscopic-assisted crestal approach). A 2.7 mm rigid endoscope was used to monitor membrane integrity after each surgical step. Bone grafting with a xenograft-platelet-rich fibrin mix was performed, followed by implant placement. Membrane perforation rates, surgical time, and bone density changes were evaluated.

**Results:**

Twenty-two implants were placed (11 in each group). Membrane perforation occurred in 33% of cases in Group 1 and 10% in Group 2 (*p* = 0.582). Group 2 had significantly longer surgical times (27.3 ± 2.00 min) compared to Group 1 (15.4 ± 2.22 min, *p* < 0.0001). Bone density increased significantly from 609.55 ± 108.71 HU postoperatively to 878.38 ± 114.60 HU at six months (*p* < 0.001). Endoscopic-assisted techniques significantly reduced perforation rates and improved surgical precision.

**Conclusions:**

The endoscopic-assisted crestal sinus lifting is a unique technique allowing visualization and assessment of membrane integrity and may enhance the safety and the predictability during membrane elevation, reducing complications such as membrane perforation. However, its benefits must be weighed against increased surgical time and cost.

## Introduction

Rehabilitation of the posterior maxilla using dental implants can be challenging due to inadequate alveolar bone height and poor bone quality [1]. The sinus floor elevation technique addresses insufficient bone height for dental implant placement and can be performed using either a lateral or crestal approach [2]. The choice depends on residual bone height and the ability to achieve adequate primary implant stability. The lateral approach, while effective for greater height gains, is invasive and carries risks such as Schneiderian membrane perforation and higher costs due to the need for a membrane to cover the osseous window [3]. To address these challenges, Summers' osteotome sinus lift (crestal approach) offers a less invasive alternative [4]. The crestal approach, often utilizing osteotomes, is minimally invasive, relatively simple, and has lower complication risks with shorter osseointegration times [5, 6]. It provides space for bone regeneration with simultaneous implant placement and bone grafting. However, it carries a risk of membrane perforation. Slow, incremental addition of graft material may reduce this risk by allowing gradual membrane detachment [7–9].

## Patients and methods

This clinical study included 20 healthy patients (8 males, 12 females; mean age 46.2 ± 8.0 years), with limited residual bone height in the posterior maxilla (4–7 mm), and adequate buccopalatal bone width (Table 1). Exclusion criteria included systemic diseases, smoking habits and sinus pathologies (Table 2). All surgeries were performed by a single experienced oral and maxillofacial surgeon with over 10 years of clinical experience in implant placement and sinus augmentation, minimizing inter-operator variability.

**Table 1:** Patient demographics

Variable	Group 1 (Conventional)	Group 2 (Endoscopic)	*p* value
Gender (M/F)	3/7	4/6	0.62
Mean age (years)	45.3 ± 8.2	47.1 3 ± 7.8	0.65

**Table 2:** Criteria for patient selection

Inclusion criteria	Exclusion criteria
Loss of one or more maxillary posterior tooth	Parafunctional habits
Patients age ≥ 21 years	Drug or alcohol abuse
Bone height was 4–7 mm	uncontrolled systemic diseases that contraindicate implant surgery
Sufficient inter-arch space and mesio-distal width	Pregnant women
	Smokers

All patients required upper posterior teeth replacement with implant-supported restorations. Radiographic assessment involved panoramic X-rays and cone-beam computed tomography (CBCT). CBCT analysis included evaluation of residual bone height, buccopalatal bone width, sinus pathology, bony septa, and precise measurement of Schneiderian membrane thickness at the planned implant sites.

All participants provided written informed consent before surgery, approving the use of clinical data for research and publication purposes. The study adhered to the ethical principles of the Declaration of Helsinki and was reviewed and approved by the Regional Ethical Review Board of Al-Azhar University.

Patients were randomly assigned to Group 1 or Group 2 using a computer-generated randomization table:

- **Group 1(non-endoscopic-guided)**: Endoscopic evaluation was performed after complete osteotomy preparation and sinus membrane elevation using osteotomes.
- **Group 2 (Endoscopic-guided)**: Endoscopic evaluation was conducted after each drilling step, osteotome malleting, and bone graft placement to monitor sinus membrane integrity.

**Fig. 1:**
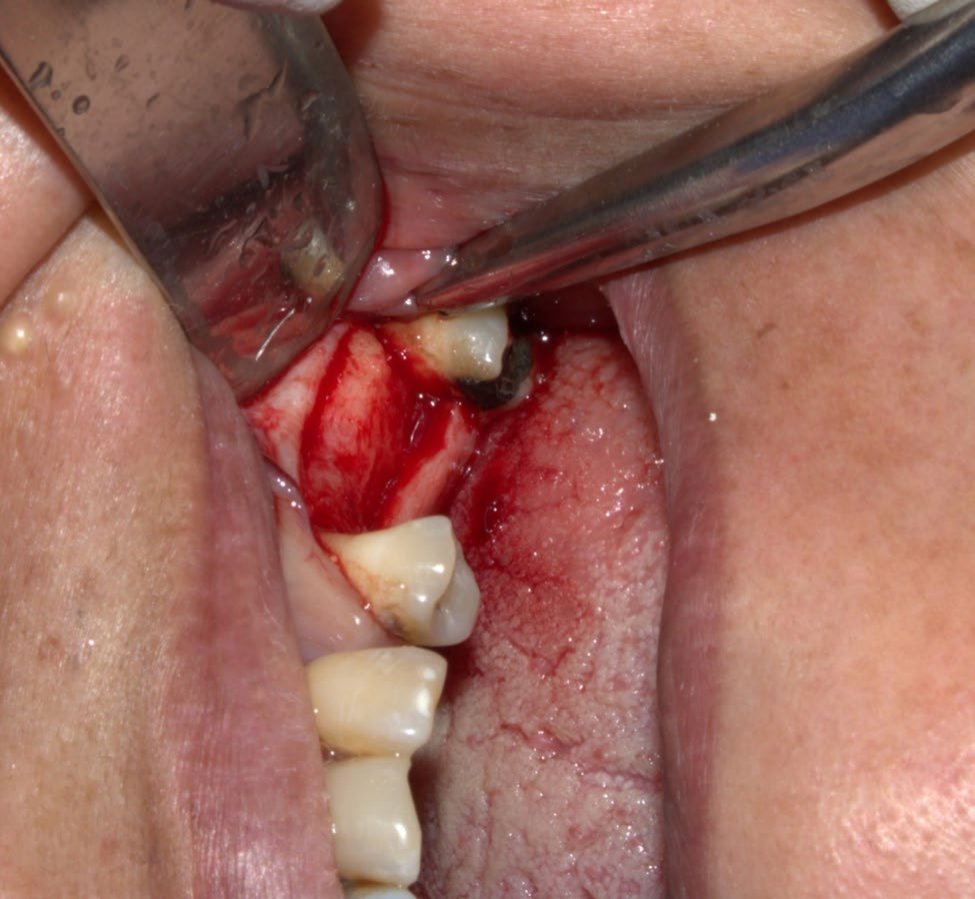
flap exposing the crest and part of the buccal bone

**Fig. 2:**
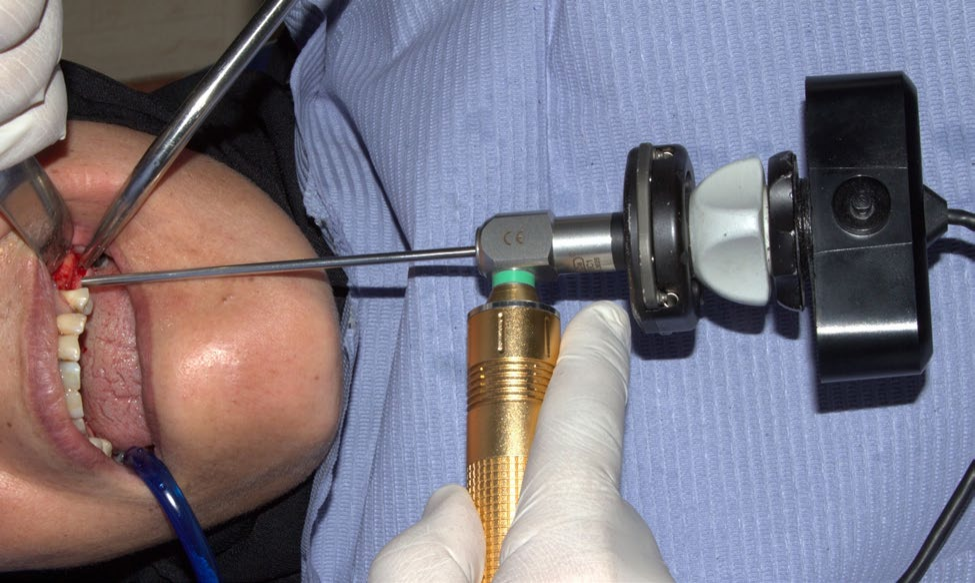
Crestal endoscopy of the sinus membrane

Under local anesthesia, a paracrestal incision was made to reflect the mucoperiosteal flap without vertical releasing incisions, facilitating bone exposure and enhanced visualization (Fig. 1). Sequential underpreparation using drills was performed under copious irrigation by omitting the last recommended drill, stopping short of the sinus membrane to prepare the osteotomy site. An endoscope was used after each drilling step to check sinus membrane integrity (Fig. 2). Sinus osteotome lifters were employed using a mallet based on the planned implant diameter and length**.** Bone quality was assessed using preoperative CBCT and intraoperative tactile feedback. Underpreparation of the osteotomy site and osteotome use condensed the native bone, effectively transforming it from D4 to D3 density. Between each osteotome insertion, a 2.7 mm rigid endoscope (zero-degree lens) was introduced into the osteotomy site from the crest for real-time monitoring the membrane integrity.

**Fig. 3:**
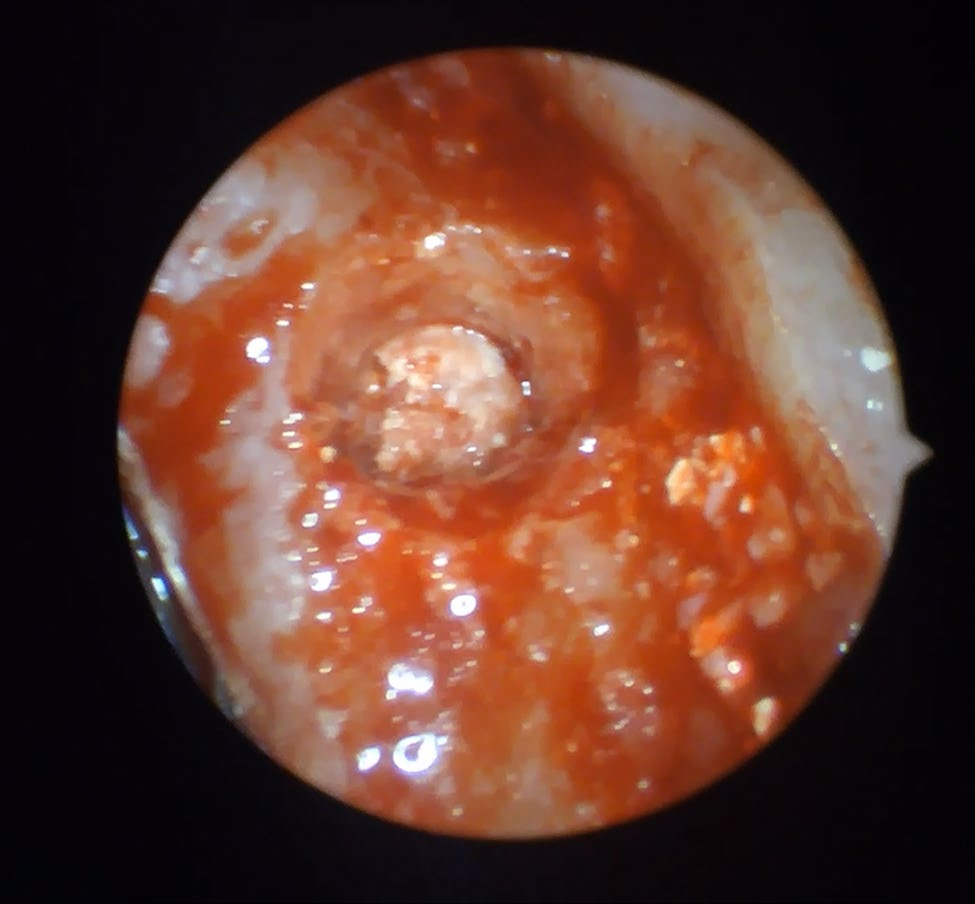
Endoscope showing bone graft apically of the osteotomy site

PRP was prepared by centrifuging the patient's blood at 3000 rpm for 15 min. In cases of membrane perforation, PRP was applied through the osteotomy site and evaluated endoscopically for closure. A xenograft-platelet-rich fibrin (PRF) mix was prepared in a bone well and inserted into the osteotomy site using an osteotome. The graft material (Bony-Tite: European Egyptian Pharmaceutical Industries, Alexandria, Egypt.) was carefully pushed apically to the desired depth, and the endoscope was used again after each insertion of the bone graft mix to ensure membrane integrity and confirm no perforation or escape of graft material into the maxillary sinus before implant placement (Fig. 3).

**Fig. 4:**
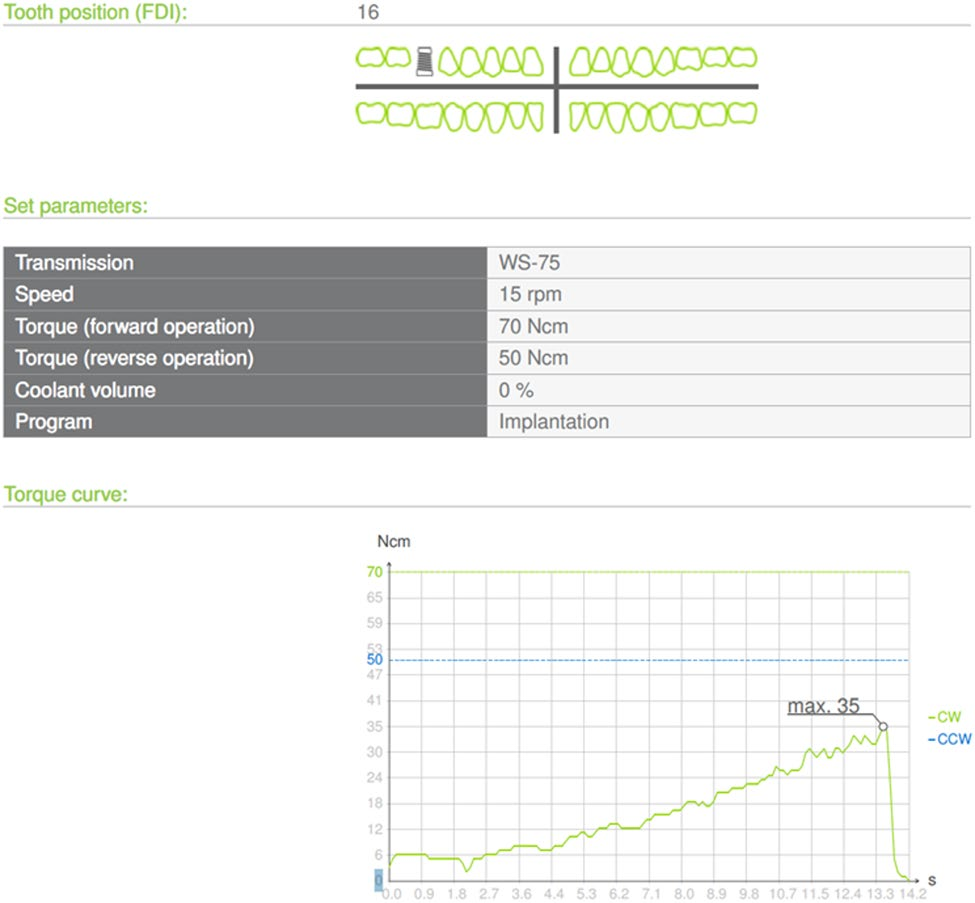
implant nsertion torque curve of dental implant

Implant diameters ranged from 3.5 to 5 mm, and lengths from 8 to 13 mm (Macros Dental Implant Systems, Ankara, Turkey). Implants were inserted using a surgical motor with a reduction dental implant handpiece set at 15 rpm and a maximum torque of 70 N-cm (Implantmed Plus, W&H Dentalwerk Burmoos GmbH, Austria). The surgical motor recorded and measured implant insertion torque values digitally. Data was stored via a USB port and exported as PDF files. Torque data were recorded as a graph showing the torque curve during implant placement (Fig. 4).

All procedures were performed under strict endoscopic guidance for real-time monitoring to ensure sinus membrane integrity. Minor membrane perforations, when detected, were managed with platelet-rich plasma (PRP) application through the prepared socket followed immediately by endoscopic evaluation for closure of the perforation. A shorter implant length was then selected than initially planned. After implant placement, the flaps were repositioned and sutured using black silk (3-0) sutures.

**Fig. 5:**
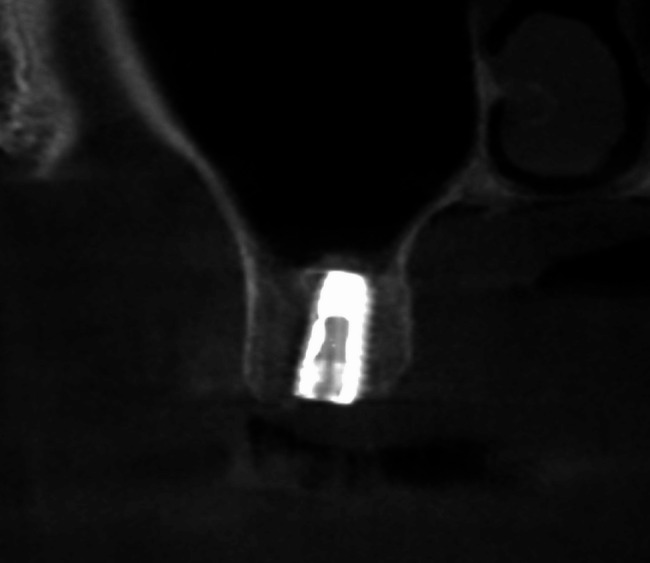
CBCT after 6 months of endoscopic assisted crestal sinus lifting

Postoperative care included antibiotics, non-steroidal anti-inflammatory drugs (NSAIDs), and detailed instructions on oral hygiene and activity restrictions. Patients were scheduled for follow-up visits seven days postoperatively and bi-monthly thereafter. CBCT scans were performed approximately six months after surgery to evaluate new bone formation, Schneiderian membrane status, and bone gained (Fig. 5). All implants achieved successful osseointegration and were loaded with final restorations. Long-term follow-ups were scheduled at regular intervals.

Data was analyzed using SPSS (Statistical Package for the Social Sciences), version 26. Qualitative data were presented as numbers and percentages. Quantitative data were tested for normality using the Shapiro–Wilk test and described as mean and standard deviation for normally distributed data, and as median and range for non-normally distributed data. The appropriate statistical test was applied according to the data type, with the following tests suggested: Student's t-test or Mann–Whitney U test for comparing two independent groups of continuous variables. The significance level was set at *P* ≤ 0.05. Perforation occurrence was clinically monitored and recorded using endoscopic evaluation through a crestal approach. Perforation occurrence was statistically compared to membrane thickness.

## Results

Twenty-two implants were placed in the posterior atrophied maxilla under endoscopic crestal monitoring for both the osteotomy site preparation and maxillary sinus membrane elevation. No cases exhibited nasal bleeding or obstruction during follow-ups. Group 1 included 10 patients (3 males, 7 females) with a mean age of 45.3 ± 8.2 years, receiving 11 implants. Group 2 included 10 patients (4 males, 6 females) with a mean age of 47.1 ± 7.8 years, receiving 11 implants.

No pain was reported during endoscopic evaluation in any patient. Using sinus lifter osteotome, the maximum insertion torque recorded was 35 N/cm, and the minimum was 11 N/cm, consistent with Type 4 bone quality.

**Fig. 6:**
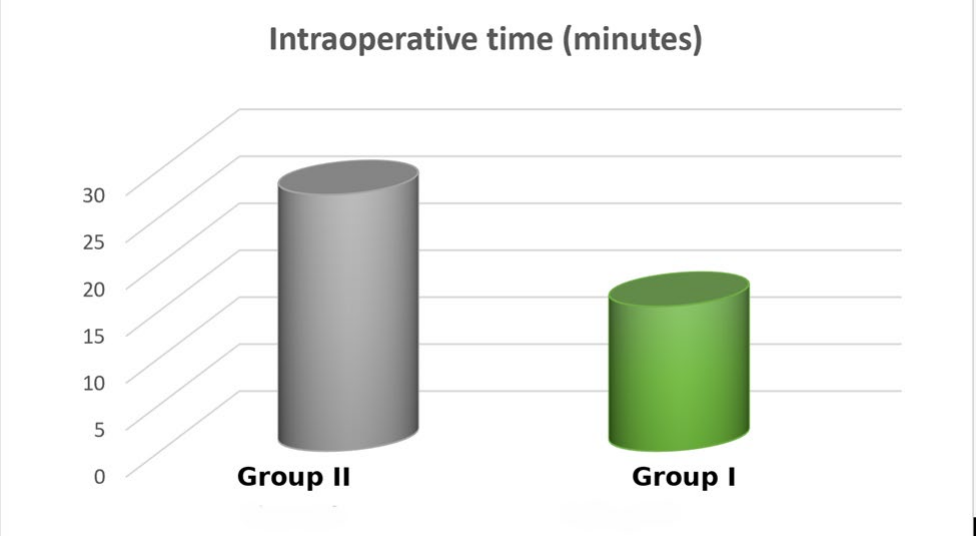
Intraoperative time means values of group 1 and group 2

The mean surgical intraoperative time for Group I was 15.4 ± 2.22 min, whereas Group II recorded longer surgical time with a mean of 27.3 ± 2.00 min (Fig. 6). Group I had significantly shorter surgical times compared to Group II (*p* < 0.0001) (Table 3).

**Table 3:** Comparison of intraoperative time between Group 1 and Group 2

Variables	Group (1)	Group (2)
Minimum (minutes)	12	24
Maximum (minutes)	19	30
Mean ± SD	15.4 ± 2.22	27.3 ± 2.00
t-value	12.58	
*P* value	< 0.0001*	

*Significant at *P* < 0.05. *Ns* non-significant at *P* > 0.05

**Fig. 7:**
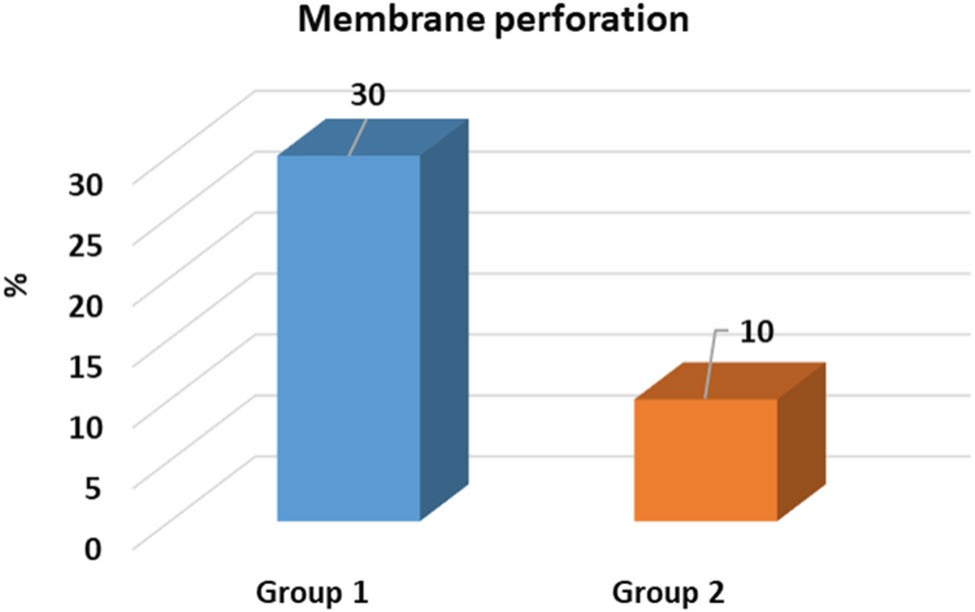
Membrane perforation among studied groups

**Fig. 8:**
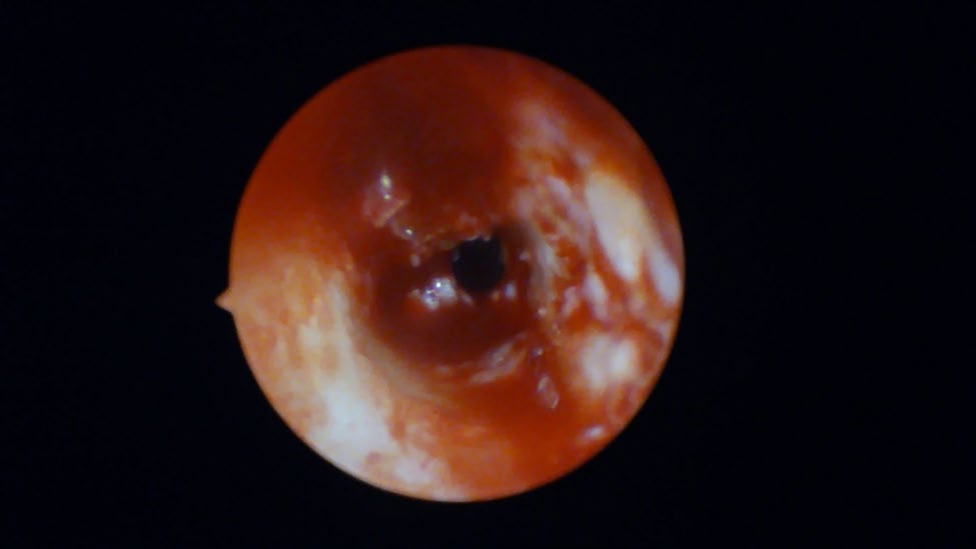
Clear perforation of the Schneiderian membrane

Membrane perforations were observed in 30% of cases (3 cases) in Group I (non-endoscopic guidance) and 10% (1 case) in Group II (*p* = 0.582) that confirms the importance of the endoscope though this difference was not statistically significant. All perforations were managed successfully using platelet-rich fibrin (PRF). Thinner membranes, with a mean thickness of 1.56 mm, were associated with a higher risk of perforation. No significant difference in membrane thickness was detected between Group I and Group II (*p* = 0.94) confirming the guided-endoscopy membrane elevation is valuable (Table 4). Thicker sinus membranes exhibited lower susceptibility to perforation. This insight underscores the value of endoscopic monitoring in reducing complications (Figs. 7, 8).

**Table 4:** comparison of residual bone height, membrane thickness, primary stability and rate of membrane perforation between studied groups

	Group (1) N = 10	Group (2) N = 10	Test of significance
Residual bone height Mean ± SD	5.14 ± 1.07	6.1 ± 0.72	t = 2.36 *p* = 0.03*
Membrane thickness median (min–max)	5(0–11)	3.85(1.6–13)	z = 0.076 *p* = 0.940
Primary stability N/cm Mean ± SD	20.80 ± 4.85	24.1 ± 6.74	t = 1.26 *p* = 0.225
Membrane perforation	3(30%)	1(10%)	FET = 1.25 *P* = 0.582

t: Student t test, Z: Mann Whitney U test, *FET* Fisher exact test

Radiographic evaluation demonstrated bone deposition around the implants, particularly near the apex, indicating successful osseointegration. CBCT scans were performed immediately after implantation and at six months postoperatively to assess changes in bone density. The mean bone density immediately after implantation was 609.55 ± 108.71 Hounsfield units (HU), which significantly increased to 878.38 ± 114.60 HU after six months (*p* < 0.001) (Table 5).

**Table 5:** Comparison of bone density immediately and after 6 months regarding time

Variables	Both groups
Immediate (HU)	609.55 ± 108.71
After 6 months (HU)	878.38 ± 114.60
t-value	5.485
*P* value	< 0.001*

*Significant at *P* < 0.05. *Ns* non-significant at *P* > 0.05. *HU* Hounsfield units

**Fig. 9:**
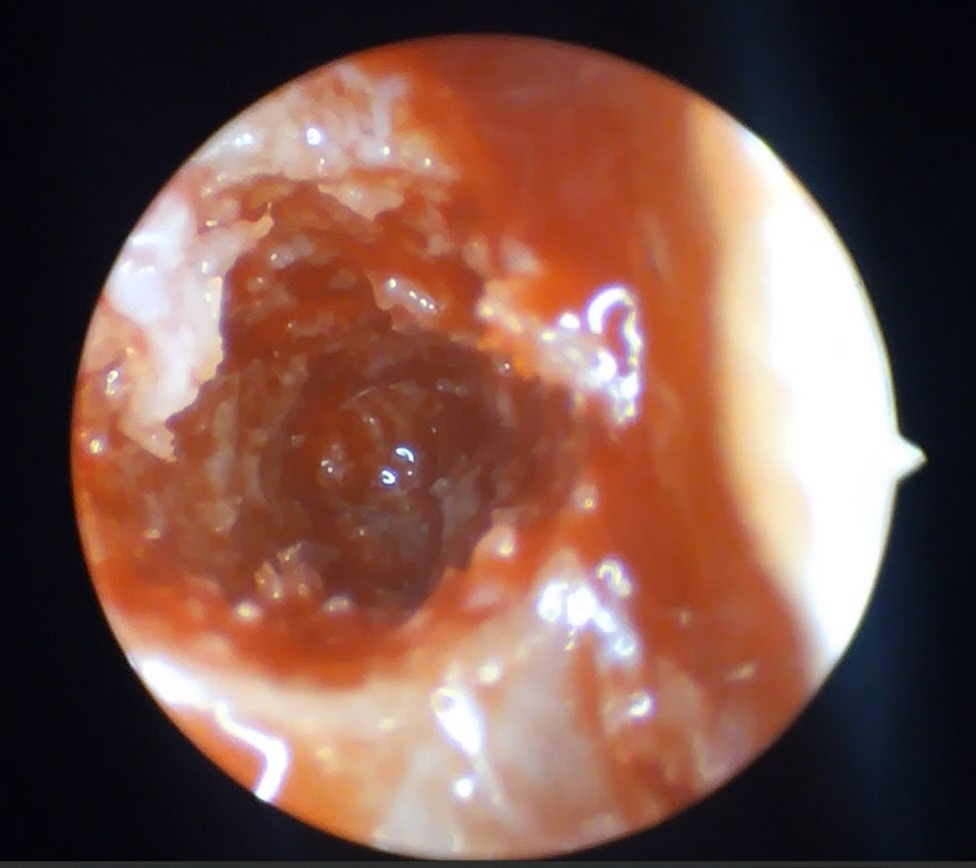
Grey shadow of the sinus membrane indicating high liability of perforation

**Fig. 10:**
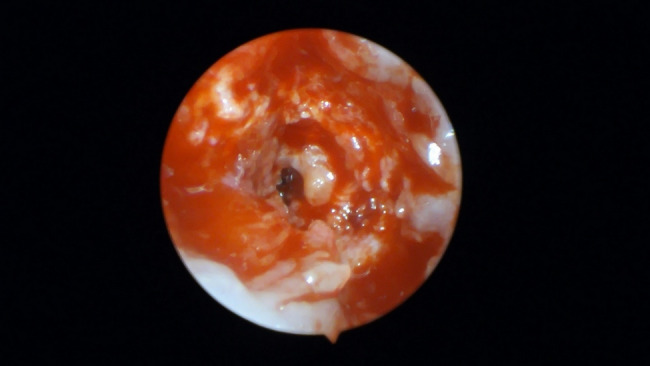
Minor hidden sinus membrane perforation

Endoscopic techniques offer long-term benefits by reducing complications such as undetected visual minor perforation through enhanced endoscopic visualization and precise minor sinus membrane evaluation (Figs. 9, 10).

## Discussion

The crestal sinus lifting technique, compared to the lateral window approach, is considered relatively straightforward and minimally invasive. It is associated with a lower risk of complications and a shorter osseointegration period, particularly when minimal drilling is used for osteotomy site preparation [4, 6, 10].

Sinus elevation using the crestal approach with osteotomes allows for controlled elevation of the Schneiderian membrane, creating a tented space that facilitates bone regeneration. This space is typically maintained by the simultaneous placement of the dental implant, often in conjunction with bone graft materials. In our study, the underpreparation of the osteotomy site followed using a mallet to drive osteotomes has been shown to enhance bone quality by condensing the native bone, effectively transforming bone from D4 to D3 density [11]. This controlled sinus membrane elevation via the osteotome technique has demonstrated a lower incidence of complications compared to other methods [12, 13].

The necessity of bone grafting in sinus floor elevation remains a subject of ongoing debate. However, clinical studies have indicated that implant survival rates do not differ significantly between cases where bone grafts are used and those where they are not [14]. Nonetheless, the osteotome technique offers several notable advantages, including reduced invasiveness, the need for smaller flaps, lower postoperative morbidity, and higher patient acceptance [15].

Despite its advantages, concerns persist regarding the extent of bone height that can be predictably elevated without risking membrane perforation during implant placement. Unlike the lateral window technique, the osteotome approach involves an internal access route, where bone augmentation is performed through the same osteotomy channel used for implant insertion. Furthermore, the osteotome method typically requires a smaller volume of graft material (1–1.5 cc) compared to the lateral window technique, which often necessitates a larger graft volume (4–6 cc) [16–18].

The use of crestal endoscopic approach is safe with minimal complications and it can precisely detect minor perforation. Moreover, crestal endoscopic evaluation seemed to be a reliable and easy method which will seriously affect clinician decision of abandoning or completing procedure following perforation detection. However, its availability as chair side equipment could be costive and technically demanding procedure [19].

The choice of graft material was not as important as the maintenance of membrane integrity as successful membrane elevation always resulted in bone formation. Bone graft may potentiate risk of membrane perforation especially at thinner areas of stretched membrane [8, 20].

Endoscope controlled sinus floor augmentation may have a lower postoperative complication rate for the transrectal procedure in patients with 4.0–7.0 mm of vertical bone height below the sinus floor [21]. The advantages offered by the magnification of endoscope gave better visualization to protect Schneiderian membrane. With endoscope, perforations of sinus membrane can be visualized; however, they cannot be avoided. The endoscopy required a higher level of surgical training and experience in endoscopic imaging [22].

The limited height gain of 3 mm of sinus floor elevation generally had reported by most clinicians, the procedure became less of a choice when the residual alveolar bone was 5–6 mm because of the concern of membrane perforation. Based on clinical findings discussed and their own clinical experience, the authors had believed that membrane perforation was not necessarily a result of large gain at height but could be more related to the hasty addition of large increments of graft material. To overcome this situation, the authors believed that slow increments of wet graft material over a long period of time might be important. This may allow time for the attachment of the membrane to be detached slowly from the sinus. This may also allow time for inflammation to occur and cause edema under sinus membrane, which will help elevation of the membrane [18, 23]. In our study, we gained up to 6 mm of bone height depending on the endoscopic evaluation of the sinus membrane after each addition of bone graft material.

With the use of endoscopy through the canine fossa into sinus cavity, Nkenke [24] was able to visualize sinus-membrane elevation and quantify the gain in height of implant sites with 18 endoscopically controlled osteotome sinus-floor elevations. The increases at height of the implants by an osteotome technique alone, up to the point where the concomitant spontaneous dissection of sinus membrane in the periphery of the elevated region stopped and the tension of sinus membrane revealed the risk of membrane perforation, was 3 mm (range 2–5 mm). Reiser, [25] had stated that using direct visualization of sinus cavity through lateral wall of nose on 16 formaldehyde-treated cadaver heads, evaluated sinus-membrane response in relation to perforation and elevated bone height (from 4 to 8 mm) with osteotome technique. The result showed that although there were six perforations out of 25 osteotome sinus-lift procedures (24% sinus-membrane perforation rate), five of the six perforations had occurred at 6–8 mm elevations and only one had occurred in the 4–5 mm elevations for osteotome technique.

Endoscopic crestal sinus lifting was a relatively new and simple technique that had shown promising results. The endoscopic crestal sinus lifting approach allowed the surgeon to precisely identify the sinus floor, evaluate remaining bone quantity, and perform augmentation with greater accuracy. Endoscopy also had provided improvement in visualization of any potential complications, such as perforations of the sinus membrane or bleeding. Although the use of endoscopy in crestal sinus lifting can increase the safety and predictability of procedure, giving better outcomes for patients, it took extra time in evaluating the sinus membrane [26, 27].

Endoscopic-assisted crestal sinus lifting further improves precision by allowing real-time visualization of the sinus membrane, enhancing safety and predictability. This approach reduces the risk of abandoning procedures due to undetected perforations. Although endoscopy is costly and requires specialized training, it provides significant benefits in terms of reduced complications such as membrane perforations and improved outcomes. In our study, endoscopic-assisted techniques reduced the incidence of membrane perforation from 33 to 10%, highlighting its potential for improving surgical outcomes. However, perforations remain possible even with magnified visualization, emphasizing the need for careful technique [28–30].

Endoscopy is valuable for high-risk cases (thin membranes, limited bone) but may not be cost-effective for routine use.

## Conclusion

Endoscopic magnification and high-resolution optics have enhanced precision and safety in crestal sinus floor augmentation. It improves visualization of the sinus membrane, allowing early detection of complications and aiding in surgical decision-making. Endoscopic-assissted crestal sinus lifting enhances the predictability of potential sinus membrane elevation, leading to better patient outcomes compared to conventional crestal sinus lifting. However, these advantages must be balanced against increased surgical time, cost, and the need for specialized training.

## Data Availability

The data supporting our findings can be requested for free at any time.
